# Supplementary material for: The Methyltransferase HemK Regulates the Virulence and Nutrient Utilization of the Phytopathogenic Bacterium Xanthomonas citri Subsp. citri
Source: Int J Mol Sci. 2022 Apr 1;23(7):3931. doi: 10.3390/ijms23073931 (PMC8999716; doi:10.3390/ijms23073931)
Supplement: Supplementary file 1 [file ijms-23-03931-s001.zip › supp-tables.pdf]

Supplementary Table S1. Differentially expressed genes of the *hemK* mutant (q-values < 0.05 and a minimum absolute |log2(Fold_change)| > 1)

Locus tag	Gene name	Gene description	Log_2Fold _change
Novel00007	-	-	-1.40
Novel00013	-	-	-1.24
Novel00014	-	-	-1.32
Novel00094	-	-	-4.64
Novel00102	-	-	-2.60
Novel00110	-	-	-1.48
Novel00116	-	-	-1.72
XAC0076	avrBs2	avirulence protein	-1.44
XAC0108	atsE	AtsE	-1.20
XAC0162	dctP	C4-dicarboxylate transport system	-1.79
XAC0163	dctQ	C4-dicarboxylate membrane transport protein	-1.20
XAC0172	XAC0172	conserved hypothetical protein	-1.01
XAC0239	XAC0239	conserved hypothetical protein	-1.06
XAC0277	XAC0277	conserved hypothetical protein	-1.58
XAC0286	avrXacE1	avirulence protein	-2.11
XAC0315	XAC0315	hypothetical protein	-1.52
XAC0330	cmfA	conditioned medium factor	-1.22
XAC0345	ilvD	dihydroxy-acid dehydratase	-1.80
XAC0346	XAC0346	degenerated cellulase	-1.05
XAC0362	pobB	phenoxybenzoate dioxygenase beta subunit	-1.03
XAC0393	hpaF	HpaF protein	-1.29
XAC0394	hrpF	HrpF protein	-2.46
XAC0395	XAC0395	hypothetical protein	-2.02
XAC0396	hpaB	HpaB protein	-2.97
XAC0397	hrpE	HrpE protein	-3.44
XAC0398	hrpD6	HrpD6 protein	-2.84
XAC0399	hrpD5	HrpD5 protein	-2.72
XAC0400	hpaA	HpaA protein	-3.10
XAC0401	hrcS	HrcS protein	-3.72
XAC0402	hrcR	HrcR protein	-3.10
XAC0403	hrcQ	HrcQ protein	-3.39
XAC0404	hpaP	HpaP protein	-2.78
XAC0405	hrcV	HrcV protein	-2.70
XAC0406	hrcU	HrcU protein	-3.15
XAC0407	hrpB1	HrpB1 protein	-3.98
XAC0408	hrpB2	HrpB2 protein	-4.39
XAC0409	hrcJ	HrcJ protein	-3.80
XAC0410	hrpB4	HrpB4 protein	-2.82
XAC0411	hrpB5	HrpB5 protein	-2.76

(*Continued on next page*)

Table S1. (*Continued from previous page*)

Locus tag	Gene name	Gene description	Log_2FoldChange
XAC0412	hrcN	HrcN protein	-2.32
XAC0413	hrpB7	HrpB7 protein	-2.20
XAC0414	hrcT	HrcT protein	-1.65
XAC0415	hrcC	HrcC protein	-1.38
XAC0416	hpa1	Hpa1 protein	-4.65
XAC0417	hpa2	Hpa2 protein	-2.55
XAC0419	XAC0419	conserved hypothetical protein && -	-1.44
XAC0435	virK	VirK protein	-2.47
XAC0465	XAC0465	metalloproteinase	-1.01
XAC0501	XAC0501	conserved hypothetical protein	-1.83
XAC0543	XAC0543	conserved hypothetical protein	-2.47
XAC0545	aroG	phospho-2-dehydro-3-deoxyheptonate aldolase%2C phe-sensitive	-1.15
XAC0552	XAC0552	endoproteinase Arg-C	-2.17
XAC0605	XAC0605	conserved hypothetical protein	-1.35
XAC0606	XAC0606	endonuclease	-1.24
XAC0612	engXCA	cellulase	-1.32
XAC0661	peh-1	endopolygalacturonase	-2.42
XAC0678	XAC0678	conserved hypothetical protein	-1.13
XAC0682	XAC0682	conserved hypothetical protein	-1.28
XAC0754	XAC0754	hypothetical protein	-2.29
XAC0795	xcp	protease	-2.19
XAC0802	XAC0802	sulfotransferase	-1.54
XAC0803	XAC0803	methyltransferase	-1.28
XAC0817	XAC0817	hypothetical protein	-1.81
XAC0878	pcaH	protocatechuate 3%2C4-dioxygenase beta chain	-1.89
XAC0879	ligA	protocatechuate degradation protein	-2.27
XAC0880	pcaQ	transcriptional regulator	-1.72
XAC0933	XAC0933	truncated xylanase	-1.02
XAC0999	cirA	colicin I receptor	-1.07
XAC1059	ard	antirestriction protein	-1.61
XAC1171	stkXac1	serine/threonine kinase	-1.62
XAC1137	prpB	carboxyphosphonoenolpyruvate phosphonomutase	-1.11
XAC1172	XAC1172	conserved hypothetical protein	-1.55
XAC1187	XAC1187	hydroxylase large subunit	-1.31
XAC1241	XAC1241	hypothetical protein	-1.91
XAC1266	hrpXct	HrpX protein	-1.24
XAC1430	dapD	2%2C3%2C4%2C5-tetrahydropyridine-2-carboxylate N-succin	-1.10
XAC1431	XAC1431	conserved hypothetical protein	-1.02

(*Continued on next page*)

Table S1. (*Continued from previous page*)

Locus tag	Gene name	Gene description	Log_2FoldChange
XAC1432	dapE	succinyl-diaminopimelate desuccinylase	-1.07
XAC1471	XAC1471	conserved hypothetical protein	-1.06
XAC1556	fucP	glucose-galactose transporter	-1.26
XAC1557	scrK	fructokinase	-1.17
XAC1666	tsr	chemotaxis protein	-1.07
XAC1820	metL	aspartokinase	-3.35
XAC1821	thrB	homoserine kinase	-3.39
XAC1823	thrC	threonine synthase	-3.60
XAC1827	XAC1827	conserved hypothetical protein	-3.16
XAC1828	hisG	ATP phosphoribosyltransferase	-2.43
XAC1829	hisD	histidinol dehydrogenase	-2.29
XAC1830	hisC	histidinol-phosphate aminotransferase	-2.36
XAC1831	hisB	imidazoleglycerolphosphate dehydratase/histidinol-phosphate phosphatase bifunctional enzyme	-2.46
XAC1832	hisH	amidotransferase	-1.90
XAC1833	hisA	phosphoribosylformimino-5- aminoimidazole carboxam	-2.21
XAC1834	hisF	cyclase	-2.26
XAC1835	hisI	phosphoribosyl-AMPcyclohydrolase/ pyrophosphatase bifunctional enzyme	-2.40
XAC1892	tsr	chemotaxis protein	-1.11
XAC1893	tsr	chemotaxis protein	-1.91
XAC1894	tsr	chemotaxis protein	-1.37
XAC1895	tsr	chemotaxis protein	-2.44
XAC1922	XAC1922	hypothetical protein	-1.07
XAC2151	yapH	YapH protein	-1.76
XAC2370	XAC2370	conserved hypothetical protein	-1.24
XAC2653	S	phage-related tail protein	-2.57
XAC2654	XAC2654	conserved hypothetical protein	-3.00
XAC2786	XAC2786	conserved hypothetical protein	-2.36
XAC2787	XAC2787	hypothetical protein	-1.37
XAC2830	fhuA	TonB-dependent receptor	-1.30
XAC2831	XAC2831	extracellular serine protease	-2.72
XAC2832	XAC2832	conserved hypothetical protein	-2.24
XAC2833	XAC2833	extracellular serine protease	-1.12
XAC2853	XAC2853	cysteine protease	-4.49
XAC2922	hrpW	HrpW protein	-2.74
XAC2976	XAC2976	conserved hypothetical protein	-1.06
XAC2990	XAC2990	hypothetical protein	-1.00

(*Continued on next page*)

Table S1. (*Continued from previous page*)

Locus tag	Gene name	Gene description	Log_2FoldChange
XAC2992	XAC2992	endoproteinase Arg-C	-1.55
XAC3014	rebB	RebB protein	-1.70
XAC3015	rebB	RebB protein	-2.13
XAC3016	rebA	RebA protein	-1.93
XAC3017	rebB	RebB protein	-2.87
XAC3018	XAC3018	hypothetical protein	-1.13
XAC3019	XAC3019	hypothetical protein	-1.03
XAC3021	XAC3021	hypothetical protein	-1.97
XAC3022	XAC3022	hypothetical protein	-1.20
XAC3085	XAC3085	conserved hypothetical protein	-1.12
XAC3087	rebB	rebB protein	-1.81
XAC3088	XAC3088	hypothetical protein	-1.28
XAC3090	XAC3090	leucin rich protein	-1.88
XAC3114	pqqG	pyrroloquinoline quinone biosynthesis protein G	-1.07
XAC3191	btuR	cob(I)alamin adenolsyltransferase	-1.37
XAC3230	XAC3230	hypothetical protein	-1.53
XAC3447	thiC	thiamine biosynthesis protein	-1.93
XAC3451	ilvC	ketol-acid reductoisomerase	-2.66
XAC3452	ilvG	acetolactate synthase isozyme II large subunit	-2.22
XAC3453	ilvM	acetolactate synthase isozyme II small subunit	-2.53
XAC3454	tdcB	threonine dehydratase catabolic	-2.14
XAC3455	leuA	2-isopropylmalate synthase	-1.30
XAC3488	suc1	sugar transporter	-1.41
XAC3489	fyuA	TonB-dependent receptor	-2.32
XAC3490	XAC3490	amylosucrase or alpha amylase	-1.90
XAC3545	XAC3545	protease	-2.18
XAC3546	xadA	outer membrane protein	-1.66
XAC3604	XAC3604	conserved hypothetical protein	-1.45
XAC3646	XAC3646	hypothetical protein	-1.25
XAC3647	pheA	chorismate mutase	-1.79
XAC3664	ompW	outer membrane protein	-1.74
XAC3666	XAC3666	hypothetical protein	-1.25
XAC3684	XAC3684	conserved hypothetical protein	-1.23
XAC3685	XAC3685	conserved hypothetical protein	-1.40
XAC3703	XAC3703	conserved hypothetical protein	-1.24
XAC3769	nucA	endonuclease precursor	-1.50
XAC3844	XAC3844	conserved hypothetical protein	-1.00
XAC3845	XAC3845	conserved hypothetical protein	-1.13
XAC3866	XAC3866	conserved hypothetical protein	-1.61
XAC3878	XAC3878	disulphide-isomerase	-1.50

(*Continued on next page*)

Table S1. (*Continued from previous page*)

Locus tag	Gene name	Gene description	Log_2FoldChange
XAC3921	ugt	glucosyltransferase	-1.21
XAC3922	entF	ATP-dependent serine activating enzyme	-1.18
XAC3963	XAC3963	tRNA-Ala	-2.24
XAC3964	XAC3964	conserved hypothetical protein	-2.89
XAC3965	XAC3965	conserved hypothetical protein	-1.67
XAC3966	XAC3966	conserved hypothetical protein	-1.12
XAC4007	XAC4007	conserved hypothetical protein	-1.03
XAC4048	iroN	TonB-dependent receptor	-1.60
XAC4063	XAC4063	hypothetical protein	-1.07
XAC4078	mgtE	Mg++ transporter	-1.72
XAC4169	mltA	transglycosylase associated protein	-1.05
XAC4327	uahA	urea amidolyase	-1.83
XAC4333	XAC4333	conserved hypothetical protein	-1.22
XAC2859	XAC2859	hypothetical protein	4.82
XAC2363	XAC2363	hypothetical protein	4.05
Novel00011	-	-	3.22
XAC2338	XAC2338	conserved hypothetical protein	2.73
XAC2749	XAC2749	methylated-DNA-protein-cysteine S-methyltransferase related protein	2.70
XAC0919	pntA	pyridine nucleotide transhydrogenase	2.67
XAC2337	cydB	cytochrome D ubiquinol oxidase subunit II	2.63
XAC3658	XAC3658	hypothetical protein	2.29
XAC0830	tauD	taurine dioxygenase	2.21
XAC2033	mobA	molybdopterin guanine dinucleotide synthase && PF12804:MobA-like NTP transferase domain	2.11
XAC0657	mreC	rod shape-determining protein	2.04
XAC2264	XAC2264	hypothetical protein	2.00
XAC1706	XAC1706	conserved hypothetical protein	1.96
XAC3255	XAC3255	hypothetical protein	1.94
XAC1639	sdeB	atrazine chlorohydrolase	1.89
Novel00097	-	PF12836:Helix-hairpin-helix motif	1.87
XAC1181	XAC1181	conserved hypothetical protein	1.85
Novel00012	-	-	1.85
XAC2951	comEA	DNA transport competence protein	1.84
XAC1980	flgH	flagellar L-ring protein	1.80
XAC1638	hutI	imidazolonepropionase	1.77
XAC1704	XAC1704	ABC transporter ATP-binding protein	1.76
XAC2558	XAC2558	excinuclease ABC subunit C homolog	1.72
XAC2608	XAC2608	VirB6 protein	1.69
XAC3354	ompW	outer membrane protein W	1.68

(*Continued on next page*)

Table S1. (*Continued from previous page*)

Locus tag	Gene name	Gene description	$Log_2FoldChange$
XAC3927	XAC3927	conserved hypothetical protein	1.66
XAC0637	hslV	ATP-dependent HslUV protease peptidase subunit HslV	1.65
XAC2528	htpG	heat shock protein G	1.64
XAC2336	cydA	cytochrome D ubiquinol oxidase subunit I	1.59
XAC0453	XAC0453	conserved hypothetical protein	1.59
XAC0505	XAC0505	conserved hypothetical protein	1.58
XAC2334	cydC	ABC transporter ATP-binding protein	1.57
XAC0638	hslU	ATP-dependent HslUV protease ATP-binding subunit HslU	1.57
XAC1300	hslR	heat shock protein 15 homolog	1.57
XAC1522	dnaK	DnaK protein	1.57
XAC3195	clpB	ATP-dependent Clp protease subunit	1.55
XAC1816	XAC1816	hemagglutinin/hemolysin-related protein	1.55
XAC2146	XAC2146	hypothetical protein	1.50
XAC1521	grpE	heat shock protein GrpE	1.48
XAC2034	XAC2034	conserved hypothetical protein	1.47
XAC2335	strW	transport protein	1.46
XAC3608	uptB	maleylacetoacetate isomerase	1.46
XAC2397	XAC2397	conserved hypothetical protein	1.45
XAC3962	XAC3962	hypothetical protein	1.43
XAC1941	fliR	flagellar biosynthetic protein	1.42
XAC3754	XAC3754	conserved hypothetical protein	1.40
XAC1635	hutU	urocanate hydratase	1.40
XAC1092	XAC1092	tRNA-Ser	1.39
XAC2312	XAC2312	conserved hypothetical protein	1.37
XAC2362	XAC2362	conserved hypothetical protein	1.34
XAC2200	XAC2200	hypothetical protein	1.33
XAC3121	fepA	TonB-dependent receptor	1.33
XAC3301	XAC3301	conserved hypothetical protein	1.32
XAC1937	flhB	flagellar protein	1.31
XAC1985	flgC	flagellar biosynthesis cell-proximal portion of basal-body rod	1.30
XAC4206	XAC4206	hypothetical protein	1.29
XAC1151	hspA	low molecular weight heat shock protein	1.28
XAC2978	ptsH	phosphotransferase system HPr enzyme	1.28
XAC3120	glk	glucose kinase	1.27
XAC1637	hutH	histidine ammonia-lyase	1.27
XAC0541	groES	10kDa chaperonin	1.27
XAC1636	hutG	formylglutamate amidohydrolase	1.26

(*Continued on next page*)

Table S1. (*Continued from previous page*)

Locus tag	Gene name	Gene description	Log2FoldChange
XAC0900	pms	peptide methionine sulfoxide reductase	1.26
XAC0627	XAC0627	conserved hypothetical protein	1.26
XAC2399	htpX	heat shock protein	1.26
XAC2742	btuB	TonB-dependent receptor	1.25
XAC1986	flgB	flagellar protein	1.25
XAC1935	flhF	flagellar protein	1.23
XAC4268	XAC4268	conserved hypothetical protein	1.22
XAC0755	XAC0755	conserved hypothetical protein	1.22
XAC0127	XAC0127	conserved hypothetical protein	1.22
XAC2863	XAC2863	hypothetical protein	1.21
XAC2375	XAC2375	conserved hypothetical protein	1.21
XAC1705	XAC1705	MFS transporter	1.20
XAC2783	trx	thioredoxin	1.19
XAC3691	XAC3691	conserved hypothetical protein	1.19
XAC0087	XAC0087	hypothetical protein	1.18
XAC3609	uptA	fumarylacetoacetate hydrolase	1.17
XAC3635	XAC3635	conserved hypothetical protein	1.16
XAC0542	groEL	60 kDa chaperonin	1.16
XAC2483	XAC2483	conserved hypothetical protein	1.16
XAC1741	recX	RecX protein	1.16
Novel00010	-	PF00118:TCP-1/cpn60 chaperonin family	1.16
XAC4216	tatC	sec-independent protein translocase	1.15
XAC1740	recA	RecA protein	1.15
XAC1984	flgD	flagellar protein	1.15
XAC1955	fliE	flagellar protein	1.15
XAC1593	XAC1593	ABC transporter ATP-binding protein	1.14
XAC2228	XAC2228	transcriptional regulator tetR family	1.14
XAC3444	btuB	TonB-dependent receptor	1.13
XAC4286	mutM	formamidopyrimidine DNA glycosylase	1.13
XAC4104	XAC4104	hypothetical protein	1.12
XAC0077	XAC0077	conserved hypothetical protein	1.12
XAC1950	fliJ	flagellar FliJ protein	1.11
XAC1936	flhA	flagellar protein	1.11
XAC1982	flgF	flagellar protein	1.11
XAC3792	ribA	riboflavin biosynthesis protein	1.11
XAC1362	nerA	GTN reductase	1.11
XAC1850	hadH2	3-hydroxyacyl-CoA dehydrogenase type II	1.10
XAC2626	fimT	fimbrial biogenesis protein	1.10
XAC2080	XAC2080	conserved hypothetical protein	1.10
XAC3755	XAC3755	conserved hypothetical protein	1.09

(*Continued on next page*)

Table S1. (*Continued from previous page*)

Locus tag	Gene name	Gene description	Log2FoldChange
XAC3363	blaI	transcriptional regulator blaI family	1.09
XAC1942	fliQ	flagellar biosynthesis	1.08
XAC3353	XAC3353	conserved hypothetical protein	1.08
XAC3961	XAC3961	transcriptional regulator tetR family	1.08
XAC3636	XAC3636	conserved hypothetical protein	1.06
XAC0691	XAC0691	conserved hypothetical protein	1.05
XAC0253	XAC0253	transcriptional regulator araC family	1.05
XAC3460	XAC3460	conserved hypothetical protein	1.05
XAC1278	XAC1278	conserved hypothetical protein	1.05
XAC2887	XAC2887	conserved hypothetical protein	1.04
XAC0134	XAC0134	beta-lactamase	1.04
XAC3823	XAC3823	conserved hypothetical protein	1.03
XAC0452	XAC0452	4-hydroxyphenylpyruvate dioxygenase	1.02
XAC0775	murE	UDP-N-acetylmuramoylalanyl-D-glutamate-2%2C6-diaminopimelate ligase domain|PF08245:Mur ligase middle domain	1.01
XAC2044	mutT	7%2C8-dihydro-8-oxoguanine-triphosphatase	1.01
XAC0772	mraW	cell division protein	1.00
XAC4279	XAC4279	conserved hypothetical protein	1.00
XAC1981	flgG	flagellar biosynthesis cell-distal portion of basal-body rod	1.00

Supplementary Table S2. List of the enriched KEGG pathways with *Xcc* genes significantly changed in the Δ*hemK* mutant

KEGGID	Description	padj	Gene Name	Log2 Fold change	Count
**1. Metabolism (37 pathways)**					
**1.1 Global and overview maps (6 pathways)**					
xac01230	Biosynthesis of amino acids	0.0003	metL/thrC/ilvC/thrB/hisI/hisD/ilvD/hisB/hisF/hisA/ilvG/hisG/tdcB/hisH/pheA/ilvM/dapD/leuA/dapE/aroG/hisC	-3.6_-1.1	21
xac01210	2-Oxocarboxylic acid metabolism	0.0821	ilvC/ilvD/ilvG/ilvM/leuA	-2.7/-1.8/-2.2/-2.5/-1.3	5
xac01110	Biosynthesis of secondary metabolites	0.9932	metL/thrC/ilvC/thrB/hisI/hisD/ilvD/hisB/hisF/hisA/ilvG/hisG/tdcB/hisH/pheA/ilvM/leuA/entF/scrK/ribA/glk/aroG/hisC	-3.6_1.3	23
xac01120	Microbial metabolism in diverse environments	0.9932	metL/thrC/thrB/dapD/uptA/XAC1187/nerA/uptB/dapE/uahA/glk	-3.6_1.5	11
xac01240	Biosynthesis of cofactors	0.9932	thiC/XAC0452/ribA/btuR	-1.9/1.0/1.1/-1.4	4
xac01200	Carbon metabolism	0.9932	tdcB/glk	-2.1/1.3	2
**1.2 Carbohydrate metabolism (11 pathways)**					
xac00500	Starch and sucrose metabolism	0.5732	XAC3490/engXCA/scrK/XAC0346/glk	-1.9/-1.3/-1.2/-1.1/1.3	5
xac00650	Butanoate metabolism	0.9932	ilvG/ilvM	-2.2/-2.5	2
xac00520	Amino sugar and nucleotide sugar metabolism	0.9932	scrK/glk	-1.2/1.3	2
xac00550	Peptidoglycan biosynthesis	0.9932	murE	1.0	1
xac00040	Pentose and glucuronate interconversions	0.9932	peh-1	-2.4	1
xac00051	Fructose and mannose metabolism	0.9932	scrK	-1.2	1
xac00640	Propanoate metabolism	0.9932	prpB	-1.1	1
xac00052	Galactose metabolism	0.9932	glk	1.3	1
xac00630	Glyoxylate and dicarboxylate metabolism	0.9932	hutG	1.3	1
xac00010	Glycolysis / Gluconeogenesis	0.9932	glk	1.3	1
xac00620	Pyruvate metabolism	0.9932	leuA	-1.3	1
**1.3 Energy metabolism (2 pathways)**					
xac00190	Oxidative	0.9932	cydB/XAC2338/cydA	2.6/2.7/1.6	3

	phosphorylation				
xac00920	Sulfur metabolism	0.9932	tauD	2.21	1
**1.4 Nucleotide metabolism (1 pathways)**					
xac00230	Purine metabolism	0.9932	XAC1187	-1.3	1
**1.5 Amino acid metabolism (10 pathways)**					
xac00340	Histidine metabolism	2.9E-10	hisI/hisD/hisB/hisF/hisA/hisG/hisH/sdeB/hutU/hutH/hutI/hisC/hutG	-2.5_1.9	13
xac00290	Valine, leucine and isoleucine biosynthesis	1.2 E-10	ilvC/ilvD/ilvG/tdcB/ilvM/leuA	-2.7/-1.8/-2.2/-2.1/-2.5/-1.3	6
xac00300	Lysine biosynthesis	0.08214	metL/dapD/murE/dapE	-3.4/-1.0/1.0/-1.1	4
xac00350	Tyrosine metabolism	0.092943	uptA/XAC0452/uptB/hisC	1.1/1.0/1.5/-2.4	4

(*Continued on next page*)

Table S2. (*Continued from previous page*)

KEGGID	Description	padj	Gene Name	Log 2Fold change	Count
xac00260	Glycine, serine and threonine metabolism	0.786303	metL/thrC/thrB/tdcB	-3.4/-3.6/-3.4/-2.1	4
xac00360	Phenylalanine metabolism	0.806129	XAC0452/hisC	1.0/-2.4	2
xac00400	Phenylalanine, tyrosine and tryptophan biosynthesis	0.930158	pheA/aroG/hisC	-1.8/-1.2/-2.4	3
xac00220	Arginine biosynthesis	0.993188	uahA	-1.8	1
xac00330	Arginine and proline metabolism	0.993188	XAC0691	1.1	1
xac00270	Cysteine and methionine metabolism	0.993188	metL	-3.4	1
**1.6 Metabolism of cofactors and vitamins (6 pathways)**					
xac00770	Pantothenate and CoA biosynthesis	0.158043	ilvC/ilvD/ilvG/ilvM	-2.7/-1.8/-2.2/-2.5/	4
xac00740	Riboflavin metabolism	0.993188	ribA	1.1	1
xac00760	Nicotinate and nicotinamide metabolism	0.993188	pntA	2.7	1
xac00790	Folate biosynthesis	0.993188	ribA/mobA	1.1/2.1	2
xac00130	Ubiquinone and other terpenoid-quinone biosynthesis	0.993188	XAC0452	1.0	1
xac00860	Porphyrin and chlorophyll metabolism	0.993188	btuR	-1.4	1
**1.7 Biosynthesis of other secondary metabolites (1 pathways)**					

xac00521	Streptomycin biosynthesis	0.993188	glk	1.3	1
**2. Genetic Information Processing (4 pathways)**					
**2.1 Folding, sorting and degradation**					
xac03060	Protein export	0.993188	tatC	1.2	1
xac03018	RNA degradation	0.497374	dnaK/-/groEL	1.6/1.2/1.2	3
**2.2 Replication and repair**					
xac03440	Homologous recombination	0.993188	recA	1.2	1
xac03410	Base excision repair	0.96327	mutM/XAC0606	1.1/-1.2	2
**3. Environmental Information Processing (3 pathways)**					
**3.1 Membrane transport (2 pathways)**					
xac03070	Bacterial secretion system	0.044174	hrcJ/hrcV/hrcQ/hrcU/hrcN/tatC/hrcC/hrpB5/hrcR/hrcS/hrcT/XAC2608	-3.8_1.7	12
xac02010	ABC transporters	0.993188	cydC/strW	1.6/1.5	2
**3.2 Signal transduction (2 pathways)**					
xac02020	Two-component system	0.993188	tsr/tsr/engXCA/tsr/tsr/tsr/XAC0346/cydB/ XAC2338/cydA	-2.4_2.6	10
**4. Cellular Processes (3 pathways)**					
**4.1 Cellular community – prokaryotes (1 pathways)**					
xac02024	Quorum sensing	0.993188	XAC3545/ribA/aroG	-2.2/1.1/-1.2	3
**4.2 Cell motility (1 pathways)**					
xac02040	Flagellar assembly	0.000594	flgH/flgD/flgC/flgG/flgB/flgF/fliJ/flhA/flhB/fliR/fliE/fliQ	1.0_1.8	12
xac02030	Bacterial chemotaxis	0.930158	tsr/tsr/tsr/tsr/tsr	-1.1/-1.1/-1.2/-1.4/-2.4	5

Supplementary Table S3. Bacterial strains and plasmids

Strains and plasmids	Relevant characteristic	Origin
X. citri subsp. *citri*		
X. citri jx-6	Wild type strain	Lab collection
ΔhemK	*hemK* mutant of *X. citri* jx-6	This study
C-*ΔhemK*	Complementation strain of *ΔhemK*, Gm	This study
E.coli		
DH5α		Lab collection
Plasmids		
pK18	Suicide vector, Gm	Lab collection
pK18-Δ*hemK*	*hemK* knock-out fragment ligated on pK18, Gm	This study
pBBR1MCS5-V5-hemK	*hemK* complementation fragment ligated on pBBR1MCS5, Gm	This study

Supplementary **Table S4**. Primers used for strains construction and qRT-PCR analysis

Primers	Sequence (5′-3′) a	Purpose
hemK-1F	CGCGGATCCTCCAGCGTCTTGATCACGC CTTC	For construction of pK18-Δ*hemK*
hemK-2R	TCAGCCGCCCTGGCCCGCAGCTGCGGC GAGATCGTCGGACAT	For construction of pK18-Δ*hemK*
hemK-3F	ATGTCCGACGATCTCGCCGCAGCTGCGG GCCAGGGCGGCTGA	For construction of pK18-Δ*hemK*
hemK-4R	GGGAAGCTTGCATGCGGCGTAGAGCGG CCGGA	For construction of pK18-Δ*hemK*
hemK-5F	ACTGTGCAGCGGTCAGCTTGCGCA	For detecting of pK18-Δ*hemK*
hemK-6R	TAGGCCAGCGCCAGCGTGGATC	For detecting of pK18-Δ*hemK*
hemK-F	TCCCCCGGGATGTCCGACGATCTCGCCG C	For construction of pBBR1MCS5-V5-hemK
hemK-R	GCATCTAGATCAGCCGCCCTGGCCCGCA GCT	For construction of pBBR1MCS5-V5-hemK
pBAD-5′S	CTGTTTCTCCATACCCGTT	A universal primer for pBBR1MCS5
M13-F	ACTGGCCGTCGTTTTAC	A universal primer for pBBR1MCS5
16s-F	TCGTATGCGGTATTAGCGTAAGT	For qPCR
16s-R	TTTGATCCTGGCTCAGAGTGAAC	For qPCR
hrpB2-F	TGGTCGATGTGCAGAACGAT	For qPCR
hrpB2-R	ATGGTCAGCTCGTGCATCAA	For qPCR
hpaA-F	GACGCTGCAACACACATACC	For qPCR
hpaA-R	GGCTTCGTTCTCTTCCTGCT	For qPCR
hrpE-F	TGTTCATGGACTTCTGGGCC	For qPCR
hrpE-R	GGTGGAATCTCTGGTGAGGC	For qPCR
virK-F	GATGTCAGTGTGAGCACCGA	For qPCR
virK-R	GGTGAAATGCGCATCGGAAA	For qPCR
hrcC-F	ACGCATCGGCCGATCTTTAT	For qPCR
hrcC-R	TCCACGGTGTTGGATCCAAG	For qPCR
avrXacE1-F	TCCACGTATCCGATTGAGCG	For qPCR
avrXacE1-R	ATGGAACTCGGTTTGCGGAT	For qPCR
avrBs2-F	GGTGTATCTGGATACCGCCG	For qPCR
avrBs2-R	TGAAGATGATGCGGCCTTGT	For qPCR
XAC3545-F	TGGTGGTCGAGCTCAAGAAC	For qPCR
XAC3545-R	CTCAGGTGCCACTGGTACTG	For qPCR
engXCA-F	TCGTTACAAGAACGTGCCGA	For qPCR
engXCA-R	GGTCGAACATACCGGGTTGT	For qPCR
XAC3490-F	GTGGATGTGGACCACGTTCT	For qPCR
XAC3490-R	GATAGGCCGTGGAATCCAGG	For qPCR
XAC2853-F	CTTTGCCCCGATGTACCTGT	For qPCR
XAC2853-R	TTTCCAGTCGTAGTTGCCCC	For qPCR
XAC1893-F	ACGCAGTTGCAACGTTTCTC	For qPCR

(*Continued on next page*)

Table S4. (*Continued from previous page*)

Primers	Sequence (5′-3′) [a]	Purpose
XAC1893-R	GTCCAGGTGCTCGAACATCA	For qPCR
XAC1666-F	CAGCATCAACGCCTTCATCG	For qPCR
XAC1666-R	ACATCACCATCAGCATCGCA	For qPCR
hisC-F	GTTGGTGTTCCTGTGTTCGC	For qPCR
hisC-R	AGATTGTCGTAACGCCCGAG	For qPCR

[a] The red ones are restriction sites.
