# Supplementary figures and images for: The Methyltransferase HemK Regulates the Virulence and Nutrient Utilization of the Phytopathogenic Bacterium Xanthomonas citri Subsp. citri
Source: Int J Mol Sci. 2022 Apr 1;23(7):3931. doi: 10.3390/ijms23073931 (PMC8999716; doi:10.3390/ijms23073931)

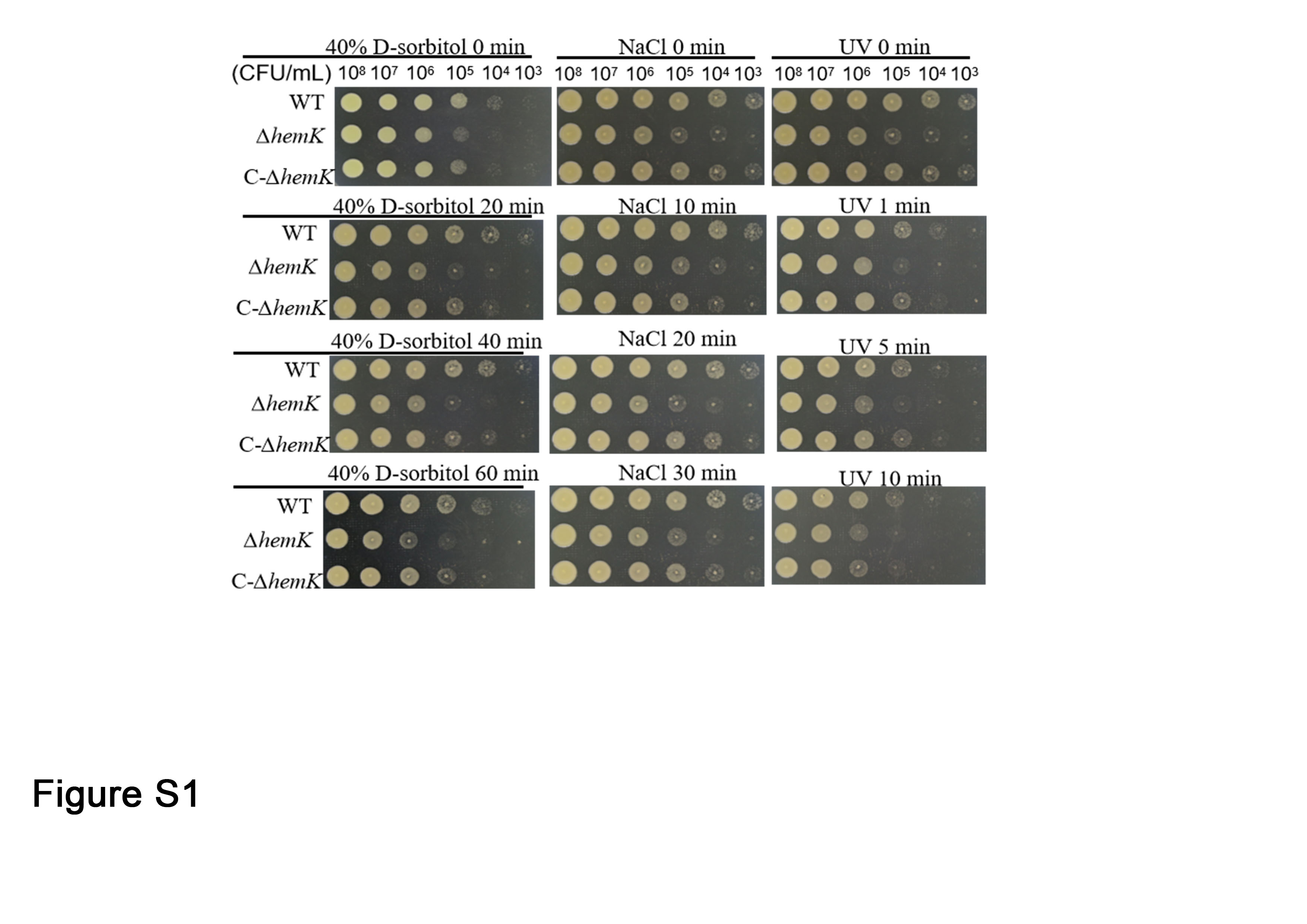

Supplement: Supplementary file 1 [file ijms-23-03931-s001.zip › Supplementary Figure S1.jpg]
